# Supplementary material for: High Normal Range of Free Thyroxine is Associated with Decreased Triglycerides and with Increased High-Density Lipoprotein Cholesterol Based on Population Representative Data
Source: J Clin Med. 2019 May 28;8(6):758. doi: 10.3390/jcm8060758 (PMC6616420; doi:10.3390/jcm8060758)
Supplement: Supplementary file 1 [file jcm-08-00758-s001.pdf]

Supplementary Table 1. Changes in lipid profiles according to TSH quartiles.

Lipid profiles	Q1 (TSH <1.55 IU/L)	Q2 (1.55-2.26 IU/L)	Q3 (2.27-3.24 IU/L)	Q4 (TSH >3.25 IU/L)	*p* for trend
**Total cholesterol (mg/dl)**					
Crude	Reference (1.0)	0.56 ± 1.84	1.11 ± 1.67	3.89 ± 1.93	0.044
Model 1	Reference (1.0)	0.52 ± 1.81	0.65 ± 1.64	3.24 ± 1.87	0.095
Model 2	Reference (1.0)	0.29 ± 1.77	0.49 ± 1.62	3.21 ± 1.87	0.094
Model 3	Reference (1.0)	0.50 ± 1.78	0.80 ± 1.63	3.63 ± 1.89	0.059
Model 4	Reference (1.0)	0.77 ± 1.78	0.67 ± 1.64	3.29 ± 1.93	0.227
**Triglyceride (mg/dl)**					
Crude	Reference (1.0)	8.35 ± 6.77	11.30 ± 7.08	4.67 ± 6.16	0.398
Model 1	Reference (1.0)	9.81 ± 6.58	13.20 ± 6.97	11.28 ± 6.00	0.119
Model 2	Reference (1.0)	8.67 ± 6.38	12.05 ± 6.74	11.16 ± 5.78	0.047
Model 3	Reference (1.0)	11.22 ± 6.26	15.66 ± 6.45	15.57 ± 5.69	0.004
Model 4	Reference (1.0)	10.57 ± 6.23	15.99 ± 6.50	16.20 ± 5.91	0.004
**HDL-cholesterol (mg/dl)**					
Crude	Reference (1.0)	0.66 ± 0.66	0.04 ± 0.64	0.35 ± 0.71	0.873
Model 1	Reference (1.0)	0.50 ± 0.62	0.24 ± 0.60	0.39 ± 0.68	0.363
Model 2	Reference (1.0)	0.63 ± 0.59	0.17 ± 0.58	0.39 ± 0.66	0.325
Model 3	Reference (1.0)	0.62 ± 0.60	0.19 ± 0.58	0.46 ± 0.66	0.273
Model 4	Reference (1.0)	0.68 ± 0.59	0.24 ± 0.59	0.50 ± 0.67	0.241
**LDL-cholesterol (mg/dl)**					
Crude	Reference (1.0)	1.77 ± 1.71	1.11 ± 1.81	2.61 ± 1.81	0.142
Model 1	Reference (1.0)	1.94 ± 1.68	1.75 ± 1.76	1.37 ± 1.76	0.448
Model 2	Reference (1.0)	2.07 ± 1.66	1.75 ± 1.76	1.37 ± 1.77	0.436
Model 3	Reference (1.0)	2.37 ± 1.65	2.14 ± 1.75	0.97 ± 1.76	0.575
Model 4	Reference (1.0)	2.02 ± 1.67	2.30 ± 1.76	0.55 ± 1.76	0.823
**Non-HDL-cholesterol (mg/dl)**					
Crude	Reference (1.0)	0.10 ± 1.88	1.15 ± 1.70	3.54 ± 1.97	0.058
Model 1	Reference (1.0)	0.02 ± 1.82	0.89 ± 1.64	3.62 ± 1.90	0.052
Model 2	Reference (1.0)	0.34 ± 1.74	0.66 ± 1.60	3.60 ± 1.88	0.046
Model 3	Reference (1.0)	0.12 ± 1.74	0.99 ± 1.61	4.09 ± 1.90	0.025
Model 4	Reference (1.0)	0.09 ± 1.75	0.90 ± 1.61	3.79 ± 1.95	0.047

The association between TSH quartiles and lipid profiles were performed by linear regression. HDL, high density lipoprotein; LDL, low density lipoprotein. Model 1: adjusted by age and sex; Model 2: Model 1 + body mass index; Model 3: Model 2 + smoking, alcohol consumption, and physical activity; Model 4: Model 3 + Urine iodine, peroxidase antibody, and diabetes mellitus.

Supplementary Table 2. Risk of dyslipidemia according to TSH quartiles.

Presence of dyslipidemia or its component	Q1 (TSH <1.55 IU/L)	Q2 (1.55-2.26 IU/L)	Q3 (2.27-3.24 IU/L)	Q4 (TSH >3.25 IU/L)	*p* for trend
**Dyslipidemia**					
Crude	Reference (1.0)	1.00 (0.80-1.24)	1.21 (0.97-1.52)	1.14 (0.91-1.41)	0.104
Model 1	Reference (1.0)	1.00 (0.80-1.25)	1.20 (0.95-1.51)	1.13 (0.90-1.40)	0.133
Model 2	Reference (1.0)	0.98 (0.78-1.24)	1.20 (0.95-1.52)	1.14 (0.91-1.43)	0.106
Model 3	Reference (1.0)	1.01 (0.80-1.27)	1.26 (0.99-1.59)	1.20 (0.95-1.52)	0.039
Model 4	Reference (1.0)	1.00 (0.79-1.26)	1.27 (1.00-1.61)	1.22 (0.96-1.54)	0.031
**Hypertriglyceridemia**					
Crude	Reference (1.0)	1.04 (0.81-1.33)	1.21 (0.94-1.55)	1.03 (0.81-1.32)	0.532
Model 1	Reference (1.0)	1.07 (0.83-1.39)	1.27 (0.98-1.65)	1.17 (0.90-1.52)	0.113
Model 2	Reference (1.0)	1.06 (0.81-1.38)	1.27 (0.97-1.67)	1.18 (0.90-1.54)	0.105
Model 3	Reference (1.0)	1.12 (0.84-1.48)	1.38 (1.04-1.82)	1.30 (0.98-1.71)	0.022
Model 4	Reference (1.0)	1.12 (0.85-1.47)	1.40 (1.06-1.86)	1.35 (1.02-1.80)	0.011
**High LDL-cholesterol**					
Crude	Reference (1.0)	1.02 (0.83-1.27)	1.00 (0.80-1.25)	1.17 (0.93-1.46)	0.224
Model 1	Reference (1.0)	1.02 (0.82-1.28)	0.97 (0.77-1.22)	1.12 (0.89-1.41)	0.469
Model 2	Reference (1.0)	1.01 (0.81-1.27)	0.97 (0.77-1.23)	1.12 (0.88-1.41)	0.465
Model 3	Reference (1.0)	1.00 (0.79-1.25)	0.95 (0.75-1.20)	1.09 (0.86-1.38)	0.603
Model 4	Reference (1.0)	1.02 (0.81-1.28)	0.94 (0.74-1.20)	1.07 (0.85-1.36)	0.745
**Non-HDL-cholesterol**					
Crude	Reference (1.0)	0.97 (0.76-1.24)	1.08 (0.85-1.37)	1.11 (0.87-1.43)	0.303
Model 1	Reference (1.0)	0.98 (0.76-1.26)	1.07 (0.83-1.37)	1.12 (0.86-1.45)	0.320
Model 2	Reference (1.0)	0.96 (0.74-1.23)	1.05 (0.82-1.36)	1.12 (0.85-1.47)	0.333
Model 3	Reference (1.0)	0.97 (0.75-1.26)	1.08 (0.84-1.40)	1.16 (0.87-1.53)	0.234
Model 4	Reference (1.0)	0.99 (0.76-1.28)	1.07 (0.83-1.38)	1.12 (0.84-1.49)	0.382

The association between TSH quartiles and dyslipidemia and component of dyslipidemia were performed by logistic regression analysis considering confounders. HDL, high density lipoprotein; LDL, low density lipoprotein. Model 1: adjusted by age and sex; Model 2: Model 1 + body mass index; Model 3: Model 2 + smoking, alcohol consumption, and physical activity; Model 4: Model 3 + Urine iodine, peroxidase antibody, and diabetes mellitus.
